# Brain connectivity in autism

**DOI:** 10.3389/fnhum.2014.00349

**Published:** 2014-06-02

**Authors:** Rajesh K. Kana, Lucina Q. Uddin, Tal Kenet, Diane Chugani, Ralph-Axel Müller

**Affiliations:** ^1^Psychology, University of Alabama at BirminghamBirmingham, AL, USA; ^2^Psychology, University of Miami, Coral GablesFL, USA; ^3^Department of Neurology, Massachusetts General HospitalBoston, MA, USA; ^4^Departments of Pediatrics and Neurology, Wayne State UniversityDetroit, MI, USA; ^5^Department of Psychology, San Diego State UniversitySan Diego, CA, USA

**Keywords:** brain connectivity, multimodal imaging methods, diffusion tensor imaging, functional connectivity, autism spectrum disorders, white matter

With the increasing prevalence of autism spectrum disorders (ASD), the pace of research aimed at understanding the neurobiology of this complex neurodevelopmental disorder has accelerated. Neuroimaging and postmortem studies have provided evidence for disruptions in functional and structural connectivity in the brains of individuals with ASD (Vissers et al., 2012). This burgeoning literature continues to struggle with methodological and conceptual issues inherent to discovering relationships between brain and behavior. While there has been considerable progress, many open questions remain. In this special topic, a collection of empirical contributions and reviews from leaders in the field attempt to synthesize and extend prior work investigating brain connectivity in autism. Multiple theoretical perspectives and neuroimaging methods are brought together with the aim of addressing outstanding questions about the nature and extent of brain connectivity aberrations in autism.

Functional connectivity magnetic resonance imaging (fcMRI), which detect correlations of the blood oxygen level dependent (BOLD) signal, provided first findings (Just et al., 2004) suggesting that the brains of individuals with ASD may exhibit reduced long-distance connectivity (Just et al., 2012). However, many more recent studies have suggested that patterns of both hypo- and hyper-connectivity can be observed in the autistic brain (Müller et al., 2011). Redcay et al., (2013) present one of the few currently available studies examining whole brain functional connectivity in ASD using graph theory and resting state fcMRI. They find that in adolescents with ASD (aged 14–20 years), a right lateral parietal region show both increased betweenness centrality and increased functional connectivity with prefrontal regions, compared with typically developing (TD) participants. Another fcMRI study by You et al. (2013) suggests that atypically increased functional connectivity in ASD may be state-dependent. The authors find that in TD children, BOLD signal correlations become reduced and more localized during sustained attention (compared to rest), whereas this is not seen in children with ASD. The study raises the important question to what extent functional overconnectivity maybe maladaptive. Delmonte et al. (2013) examine fronto-striatal circuitry in adolescents and young adults with ASD, again finding atypically increased functional connectivity—consistent with and expanding upon an earlier study (Di Martino et al., 2011). Using independent component analysis in order to identify subnetworks within the default mode network, Starck et al. (2013) report decreased connectivity between anterior and posterior default mode subnetworks in adolescents with ASD.

Whereas the studies mentioned above focus on long-range connectivity, relatively little is known from fcMRI about local connectivity. The article by Maximo et al. (2013) uses a regional homogeneity approach to reveal local overconnectivity in posterior occipital and temporal cortices alongside local underconnectivity in posterior cingulate and medial prefrontal regions in adolescents with ASD.

The fcMRI studies described above suggest that differential findings are not only region- or network-specific (Redcay et al., 2013 and Delmonte et al., 2013 vs. Starck et al., 2013), but also state-specific (You et al., 2013). An important additional aspect is discussed in a review by Uddin et al. (2013), who suggest that research on brain connectivity in autism should be placed in a developmental framework in order to more precisely pinpoint the sources of age-related group differences in functional connectivity. In their review, the authors summarize recent evidence suggesting that at younger ages closer to disorder onset, the brains of children with ASD are hyper-connected in comparison with TD controls. Keehn and colleagues provide further empirical evidence in support of this claim. They used functional near-infrared spectroscopy to examine brain connectivity in infants in the first year of life who are at high risk for developing autism. They report that at 3 months, high-risk infants showed increased connectivity compared to low-risk infants, and that between 6 and 9 months these group differences disappear and even reverse in direction (Keehn et al., 2013). Another study by Padmanabhan and colleagues examine striatal functional connectivity in a relatively large ASD and TD samples (ages 8–36 years), using resting state functional MRI. Aside from a main group effect (increased connectivity with parietal and decreased connectivity with frontal areas in ASD), they identify numerous regions in cerebellum and temporal lobe showing age-related increases of functional connectivity with striatal seeds in TD children and adults, contrasted by decreases in ASD. These findings highlight the importance of studying autism across the lifespan using multimodal neuroimaging approaches.

In addition to age-related factors that may contribute to the conflicting hypo- vs. hyper-connectivity results in the literature, several methodological factors are beginning to be identified. It is now understood that group differences in functional connectivity studies can be dramatically affected by methodological details (Jones et al., 2010; Nair et al., 2014), as for example the thorough treatment of head motion even in the sub-millimeter range (Power et al., 2012, 2014). Gotts and colleagues show how a related issue, i.e., fluctuations of the whole brain (global) signal across time, and its treatment in data preprocessing can influence the pattern of group differences observed. They find that the common practice of global signal regression can alter the location and direction of connectivity differences, obscuring neural findings. The empirical contributions by Starck et al. in this special issue carefully address these methodological concerns by explicitly characterizing and censoring motion artifacts to validate the robustness of their connectivity findings.

In contrast to the high spatial but low temporal resolution of functional connectivity MRI, MEG (magnetoencephalography) and EEG (electroencephalography) enable the measurement of functional connectivity with high temporal resolution and medium level spatial resolution. An additional advantage of these techniques is that they are not susceptible to motion artifacts that would confound connectivity results in the same way as fcMRI. Using EEG, Coben and colleagues (2014) propose a theory of mixed under- and over-connectivity in ASD, based on EEG data supporting both types of effects in ASD. The authors emphasize the use of more advanced statistical approaches to EEG coherence analysis, and discuss three different forms of multivariate connectivity analysis. In parallel, using MEG, Buard and colleagues (2013) investigate the differences in low frequency and high frequency oscillatory power in participants with ASD and their first degree relatives. They also find mixed results, with differing patterns of abnormalities in ASD across different frequency bands, opening the door to interesting potential mechanistic interpretations.

While functional connectivity studies of autism continue to reveal nuanced patterns of hypo- and hyper-connectivity associated with the disorder, studies of white matter connectivity using diffusion tensor imaging (DTI) and tractography provide complementary metrics. However, only few studies to date have combined functional and anatomical connectivity findings (Mueller et al., 2013; Nair et al., 2013). Delmonte and colleagues, despite detecting functional overconnectivity between striatum and frontal cortex during resting state, find no group differences in structural connectivity in corresponding fronto-striatal tracts, using DTI. The authors suggest that hyperconnectivity within certain circuits may be a reflection of complex functional reorganization in autism. Another study by Lewis and colleagues (2013) examines the potential impact of brain overgrowth in autism on conduction delays and long-distance connectivity, using DTI. They find network efficiency in adults with autism to be inversely correlated with intracranial volume, and suggest that the reduction in efficient connectivity in autism may be due to early brain overgrowth. Schaer and colleagues (2013) address the issue of connectivity by examining changes in cortical folding, comparing a local gyrification index with connectivity indices from DTI. This approach is based upon Van Essen's theory that mechanical tension exerted on long connections shapes cortical folds (Van Essen, 1997). While they do not observe a relationship between long-range connectivity and gyral patterns, they observe a higher gyrification index in ASD participants with higher short-range connectivity. McGrath and colleagues use a multimodal neuroimaging approach (functional MRI and High Angular Diffusion MRI) to examine the relationship between abnormal functional connectivity in a visuospatial task in autism and the integrity of corresponding white matter tracts. They find altered white matter microstructure to be related to disruptions in functional connectivity during visuospatial processing, especially in connections between left occipital lobe and five paired regions in the left hemisphere (caudate head, caudate body, uncus, thalamus, and cuneus). While findings from fcMRI and DTI do not always correspond in obvious ways (see Delmonte et al., 2013) the studies described above highlight the importance of multimodal neuroimaging approaches for a more comprehensive understanding of brain network abnormalities in ASD.

An important aspect of understanding the neurobiology of autism is to test the utility of the findings in aiding the diagnostic process, which may establish such findings as neural signatures or biomarkers. In a machine learning approach, Nielson and colleagues use a large fMRI autism database, the Autism Brain Imaging Data Exchange (ABIDE) (Di Martino et al., 2013), to classify participants with ASD from TD participants based on functional connectivity features. This study uses resting state functional connectivity data obtained from 964 participants across 16 international sites. Diagnostic classification accuracy in this study is 60% overall, disappointingly hovering just above chance. The authors suggest that additional sources of variability with use of multisite data are likely to blame, indicating a need for standardized data acquisition protocols. Their results may also indicate advantages of longer fMRI acquisition times. While this multisite study shows relatively low classification accuracy, Deshpande and colleagues (2013) use different connectivity measures, obtained from an fMRI study of theory-of-mind, in a classification analysis. They report that effective connectivity differences across 19 paths in the brain classify participants with autism from typical controls with 95% accuracy. A couple of interesting aspects of this study are: (1) While functional connectivity studies of autism are abundant, there are only a handful of studies examining effective connectivity (the causal influence of one brain area on another). This study presents differences in effective connectivity between autism and control participants. (2) The authors use different indices of connectivity (functional, effective, and white matter integrity) in their classification analysis and find effective connectivity results classifying the two groups with the highest level of accuracy. Connectivity-based pattern classification studies, with larger sample size and multiple indices, can provide valuable insight in identifying reliable neural markers of autism.

In a comprehensive review, McFadden and Minshew (2013) examine the findings of brain connectivity in autism and their underlying structural and genetic bases. Their review points to widespread abnormalities during different stages of brain development to be critical in altered brain connectivity in autism. They suggest that a relatively consistent finding involving excess of interstitial neurons may be a function of a general over-proliferation of cortical neurons or a reflection of aberrant axonal and/or synaptic connectivity during fetal life causing a subsequent failure of appropriate developmental apoptosis. This review emphasizes that axonal abnormalities and their underlying genetic bases may be critical for characterizing the neurobiology of ASD. Similarly, Zikopoulos and Barbas focus their review of postmortem microscopic changes on axonal pathology in ASD. Their findings show a complex pattern of fewer large myelinated axons and increased numbers of thin myelinated axons in superficial white matter in anterior cingulate cortex, no change in lateral prefrontal cortex, and decreased thin myelinated axons in orbital frontal cortex. These results are consistent with the notion of regionally varying patterns of hypo- and hyper-connectivity associated with ASD, as discussed above. However, knowledge of brain anomalies at the cellular level in ASD is hampered by a lack of *in vivo* imaging techniques that can detect cytoarchitectonic changes. The study by Jeong and colleagues uses a sophisticated analysis of diffusion weighted MRI data in order to detect connectivity changes in the cerebellum related to Purkinje cell loss, as known from postmortem studies. They find evidence that tracts between cerebellar cortex and dentate nuclei (i.e., axonal efferents from Purkinje cells) are compromised in children with ASD, suggesting that *in vivo* diffusion weighted MRI can generate complementary evidence in support of cellular findings from the postmortem literature.

Returning to the basic questions regarding brain network connectivity in ASD raised in the initial announcement, the contributions to this Research Topic underline the need for differentiated interpretations of functional connectivity findings that consider the specificity of networks and cognitive states under investigation and the exact preprocessing pipelines and analysis tools implemented. The need for electrophysiological studies that provide a window onto the dynamic aspects of network connectivity is further emphasized by several contributions, as is the need for multimodal investigations that combine assays of functional and anatomical connectivity. The developmental trajectory of brain connectivity and the classification potential of different connectivity measures are important topics that are investigated by different studies. Finally, several articles contribute to a better understanding of the links between cellular abnormalities in autistic cortex (both cerebral and cerebellar) and disturbances in network connectivity.

## Conflict of interest statement

The authors declare that the research was conducted in the absence of any commercial or financial relationships that could be construed as a potential conflict of interest.
